# Transcriptomic Profiling Reveals METTL26 as a Potential Biomarker in Head and Neck Squamous Cell Carcinoma with Independent Validation in Oral Squamous Cell Carcinoma

**DOI:** 10.3390/medsci14060674

**Published:** 2026-10-15

**Authors:** Rossella Rotondo, Alessandro Polizzi, Sabrina Donati Zeppa, Gaetano Isola, Roberto Campagna, Giovanni Tossetta

**Affiliations:** 1 Department for the Promotion of Human Science and Quality of Life, San Raffaele Roma University, Via di Val Cannuta, 247, 00166 Rome, Italy; rossella.rotondo@uniroma5.it (R.R.); sabrina.donatizeppa@uniroma5.it (S.D.Z.); giovanni.tossetta@uniroma5.it (G.T.); 2 IRCCS San Raffaele Roma, 03043 Cassino, Italy; 3 Department of General Surgery and Surgical-Medical Specialties, School of Dentistry, University of Catania, 95123 Catania, Italy; alessandro.polizzi@phd.unict.it (A.P.); gaetano.isola@unict.it (G.I.)

**Keywords:** METTL26, methyltransferase-like 26, head and neck squamous cell carcinoma, HNSCC, oral squamous cell carcinoma, OSCC, KEGG, STRING, TCGA-HNSC, Tdark

## Abstract

**Background:** Methyltransferase-like 26 (METTL26) is a poorly characterized member of the METTL family that has been included in previous pan-cancer analyses, but its disease-specific clinical and molecular relevance in head and neck squamous cell carcinoma (HNSCC) and oral squamous cell carcinoma (OSCC) remains poorly defined. The aim of this study was to investigate METTL26 expression and its potential clinical and biological relevance in HNSCC, with particular attention to OSCC. **Methods:** The TCGA-HNSC dataset was used as a discovery dataset. METTL26 expression and pathological stage were evaluated using GEPIA3, whereas survival associations were analyzed in R. Tumor microenvironment associations were investigated using TIMER3. Genes co-expressed with METTL26 were subjected to Gene Ontology (GO) and Kyoto Encyclopedia of Genes and Genomes (KEGG) enrichment analyses, while protein interaction networks were explored using STRING. These discovery analyses were performed using the heterogeneous TCGA-HNSC cohort, whereas two independent OSCC cohorts (GSE37991 and GSE30784) were primarily used to validate differential expression. **Results:** METTL26 expression was significantly increased in TCGA-HNSC compared to normal tissues and was associated with pathological stage. High METTL26 expression showed a non-significant trend toward poorer OS and PFI and was not independently associated with either outcome after multivariable adjustment. A positive association with cancer-associated fibroblast (CAF) abundance was detected using TIDE, whereas an inverse association with endothelial cell abundance was observed across multiple algorithms. Functional analysis showed enrichment of mitochondrial gene expression, mitochondrial translation, ribosomal functions, oxidative phosphorylation, and reactive oxygen species-related pathways. METTL26 was also positively correlated with several genes involved in therapy response such as PTOV1, MIF, BAD, and EIF3C. Importantly, METTL26 upregulation was independently confirmed in both OSCC cohorts, GSE37991 and GSE30784. **Conclusions:** METTL26 upregulation was independently confirmed in two OSCC cohorts, supporting its potential relevance as an expression biomarker. In contrast, the observed associations with pathological stage, mitochondrial and redox-related programs, stromal composition, and therapy-response-related genes should be considered exploratory and hypothesis-generating and require independent and functional validation.

## 1. Introduction

Head and neck squamous cell carcinoma (HNSCC) comprises a heterogeneous group of malignancies arising from the squamous epithelium of the upper aerodigestive tract, including the oral cavity, pharynx, and larynx. HNSCC represents one of the most common malignancies worldwide and is associated with substantial morbidity and mortality [1,2]. Tobacco smoking and alcohol consumption are the major established risk factors, particularly for tumors arising in the oral cavity and larynx, whereas infection with high-risk human papillomavirus (HPV), especially HPV16, plays a major etiological role in a subset of oropharyngeal cancers [1]. Despite significant advances in surgery, radiotherapy, chemotherapy, targeted therapy, and immunotherapy, the prognosis of patients with locally advanced or recurrent/metastatic HNSCC remains poor. Moreover, the marked molecular and clinical heterogeneity of HNSCC contributes to substantial differences in disease progression and therapeutic response among patients. Therefore, the identification of novel molecular biomarkers capable of improving patient stratification, prognostic assessment, and therapeutic personalization remains an important objective in HNSCC research [3,4].

Oral squamous cell carcinoma (OSCC) accounts for about 40% of all HNSCC cases globally, making the oral cavity one of the most commonly affected anatomic sites [5,6,7]. The main risk factors for OSCC include tobacco and alcohol consumption, betel quid and areca nut chewing in specific geographical regions, as well as several additional environmental and molecular factors [8]. Unlike oropharyngeal squamous cell carcinoma, HPV infection appears to have a more limited etiological contribution to cancers arising in the oral cavity [8]. OSCC is characterized by considerable biological heterogeneity and frequently exhibits local invasion, cervical lymph node metastasis, recurrence, and resistance to conventional treatments. Although histopathological evaluation and TNM staging remain essential for clinical management, patients presenting with apparently similar clinicopathological characteristics may experience markedly different clinical outcomes. Consequently, considerable effort has been devoted to identifying molecular biomarkers that may complement conventional parameters and provide additional information on tumor aggressiveness, prognosis, and treatment response [9,10].

Among the molecular mechanisms increasingly implicated in cancer biology, methylation-dependent regulation has emerged as an important determinant of gene expression, protein function, cellular metabolism, and tumor progression [11,12,13,14,15]. Methyltransferase-like (METTL) proteins constitute a structurally related family of S-adenosylmethionine-dependent methyltransferases capable of modifying different substrates, including RNA, proteins, and other biomolecules [16,17]. Several METTL family members have been extensively investigated in cancer and have been implicated in tumor cell proliferation, survival, invasion, metastatic dissemination, metabolic reprogramming, and response to anticancer therapies [18,19]. Moreover, increasing evidence indicates that METTL-dependent regulatory mechanisms may also be relevant to the development and progression of head and neck malignancies [20].

Methyltransferase-like 26 (METTL26), previously known as C16orf13, is a protein-coding member of the METTL family that contains a predicted S-adenosylmethionine-dependent methyltransferase domain. Although several METTL proteins have been implicated in the regulation of cancer-associated processes, the biological function, substrate specificity, and possible involvement of METTL26 in tumor biology remain largely unknown. This lack of functional information represents an important knowledge gap and raises the question of whether METTL26 may nevertheless show cancer-associated alterations. Proteomic studies have confirmed METTL26 expression in human tissues and suggested possible associations with cellular protein networks, although its precise biological role remains to be established [21]. Previous evidence has suggested a potential involvement of METTL26 in cancer. In a multi-omics analysis of 34 METTL family members across human malignancies, Campeanu et al. reported METTL26 dysregulation in selected tumor types, identified clinical associations, and suggested a possible involvement of METTL26-associated proteins in vacuolar transport [22]. However, METTL26 was not the primary focus of that study, and a dedicated characterization of its clinical and molecular relevance in HNSCC and OSCC has not been reported. Thus, investigating METTL26 expression and its associations with clinical features, tumor microenvironment composition, and molecular programs in HNSCC, together with independent validation in OSCC cohorts, may provide additional insights into its potential relevance in these malignancies.

The present study was designed as an exploratory investigation to determine whether METTL26 is differentially expressed in HNSCC/OSCC and whether its expression is associated with clinical features and specific molecular programs that could provide a rationale for subsequent functional studies. Accordingly, the present study addressed three distinct questions: first, whether METTL26 is differentially expressed in TCGA-HNSC and whether this alteration can be independently validated in OSCC; second, whether METTL26 expression is associated with pathological stage and patient outcome; and third, whether METTL26 expression is associated with specific molecular pathways and tumor-microenvironment features that may generate hypotheses regarding its possible biological involvement in HNSCC. These analyses were intended to provide an initial framework for subsequent experimental characterization of METTL26 rather than to establish a direct functional or causal role.

## 2. Materials and Methods

### 2.1. METTL26 Expression in TCGA-HNSC

Differences in METTL26 expression between TCGA-HNSC tumor samples (N = 520) and non-cancerous tissues (TCGA adjacent normal tissues supplemented with GTEx normal tissues; N = 44) were investigated using Gene Expression Profiling Interactive Analysis version 3 (GEPIA3) (https://gepia3.bioinfoliu.com accessed on 10 June 2026) tool [23]. The association between METTL26 expression and pathological stage in TCGA-HNSC was evaluated using the “Stage Plot” function of the “Expression Analysis” module of GEPIA3. The analysis included Stage I (N = 27), Stage II (N = 81), Stage III (N = 93), and Stage IV (N = 318) cases. The statistical test performed by GEPIA3 for stage analysis was one-way ANOVA.

Overall survival (OS) and progression-free interval (PFI) analyses were performed in R (v. 4.6.1), retrieving the RNA-sequencing and clinical data of the TCGA-HNSC cohort from Genomic Data Commons (GDC) using the TCGAbiolinks (v. 2.40.0) package. Expression values were log2(TPM + 1) transformed before subsequent analyses.

For Kaplan–Meier analysis, patients were dichotomized into METTL26-low and METTL26-high groups according to the median METTL26 expression value across the TCGA-HNSC cohort. Survival curves were compared using the log-rank test. PFI data were obtained from the TCGA-Clinical data resource (TCGA-CDR). Cox proportional hazards regression models were used to estimate hazard ratios (HRs) and corresponding 95% confidence intervals (CIs) for both OS and PFI. Multivariable Cox regression analyses were performed adjusting the outcome for age at diagnosis, sex, clinical stage, and anatomical tumor site. HPV-stratified Cox regression analyses were performed by including the final_HPV_status variable as a stratification factor using strata (final_HPV_status); therefore, separate baseline hazards were allowed for HPV-status groups, while the coefficients of METTL26 expression and the other covariates were estimated across strata. The analysis included 243 HPV-negative and 36 HPV-positive patients.

Cox regression analyses were performed using the survival (v. 3.8.6) package, Kaplan–Meier plots were generated using survminer (v. 0.5.2), and forest plots were generated using broom (v. 1.0.13) and ggplot2 (v. 4.0.3) packages. All statistical tests were considered statistically significant at *p* < 0.05.

### 2.2. Tumor Microenvironment Cell Infiltration Analysis

The “Immune” module of Tumor Immune Estimation Resource version 3 (TIMER3) tool (https://compbio.cn/timer3 accessed on 14 June 2026) was used to evaluate the correlations between METTL26 expression and estimated tumor-microenvironment cell abundance [24]. The following algorithms were used: TIDE, EPIC, XCELL, MCPCOUNTER, and CON-SENSUS_TME. Correlations were evaluated using tumor-purity-adjusted Spearman correlation coefficients, as implemented in TIMER3. Heatmaps and scatter plots were generated to illustrate the associations between METTL26 expression and the estimated abundance of the analyzed cell types.

### 2.3. METTL26-Related Gene Enrichment Analysis

The GEPIA3 “Expression network” module was used to identify the top 100 genes correlated with METTL26 from the TCGA dataset.

Gene Ontology (GO) and Kyoto Encyclopedia of Genes and Genomes (KEGG) pathway enrichment analyses were performed in R (version 4.6.1) using the clusterProfiler (4.20.0) package, together with org.Hs.eg.db (v. 3.23.1) for human gene annotation. Gene symbols were converted to Entrez Gene identifiers using the bitr function. Over-representation analysis was performed separately for the three GO ontologies, namely Biological Process (BP), Cellular Component (CC), and Molecular Function (MF), using the enrichGO function. Enrichment significance was assessed using the Benjamini–Hochberg method for multiple-testing correction, with both the nominal *p*-value and adjusted *q*-value thresholds set at 0.05.

KEGG pathway enrichment analysis was performed using the enrichKEGG function. Benjamini–Hochberg (BH) correction was applied, using *p* < 0.05 and *q* < 0.05 as significance thresholds. The significantly enriched pathways were ranked according to statistical significance and visualized using both bar plots and bubble plots.

### 2.4. Protein–Protein Interaction Analysis of METTL26-Related Genes

We used the STRING database (https://cn.string-db.org accessed on 27 June 2026) to identify the protein–protein interaction network of the top 100 genes correlated with METTL26 expression identified using GEPIA3, using default settings, including a minimum required interaction score of 0.400 (medium confidence), and retaining all STRING evidence channels [25].

### 2.5. Validation of METTL26 Expression in Independent Cohorts

Validation of METTL26 expression was performed in two independent publicly available Gene Expression Omnibus (GEO) datasets, GSE30784 and GSE37991, using R. The characteristics of the GSE30784 and GSE37991 datasets have been reported in Table 1. GEO expression matrices and associated sample metadata were retrieved using the GEOquery (v. 2.80.0) package. Differential expression analyses were performed using limma. Empirical Bayes moderation was applied, and *p*-values were adjusted across the entire transcriptome using the Benjamini–Hochberg procedure. Graphical representations were generated using ggplot2 (v. 4.0.3).

For GSE30784, the expression values deposited in GEO had already been processed and normalized using the gcRMA algorithm. Accordingly, these normalized expression values were used directly for the subsequent analysis. Probe-to-gene mapping identified six probe sets annotated to METTL26 (1554466_a_at, 227378_x_at, 227380_x_at, 228114_x_at, 228118_x_at, and 230851_x_at). For the gene-level validation, probe set 1554466_a_at was used. The remaining five probe sets carry the Affymetrix “_x_at” designation, indicating potential cross-hybridization, and were therefore not used to represent METTL26 expression in the final comparison. Samples were classified into three groups: normal oral tissue (control; N = 45), oral dysplasia (N = 17), and OSCC (N = 167). Differential expression was assessed using the limma (v. 3.68.5) package in R. A linear model was fitted using tissue category as a three-level factor, and three pairwise contrasts were evaluated: OSCC versus normal tissue, dysplasia versus normal tissue, and OSCC versus dysplasia. Moderated statistics were subsequently obtained using the empirical Bayes procedure implemented in limma.

For GSE37991, the expression values provided by GEO had already undergone quantile normalization. Since these processed intensities were provided on a linear scale, they were log2-transformed in R before statistical analysis; no further normalization was performed. Two probes corresponding to METTL26 were identified (ILMN_1774990 and ILMN_2369826). In the original platform annotation, these probes are associated with the historical METTL26 annotations MGC13114/C16orf13 and with RefSeq transcripts corresponding to METTL26. ILMN_1774990 was retained for the gene-level analysis because it showed a substantially higher overall signal across the samples than ILMN_2369826, thereby providing a more robust representation of METTL26 expression. Multiple probes were therefore not averaged. In addition, because GSE37991 contains matched tumor and adjacent non-tumor samples, the paired study design was incorporated into the limma analysis by including patient identity in the linear model together with tissue status.

## 3. Results

### 3.1. METTL26 Expression in Normal and HNSC Tissues

**Figure 1:**
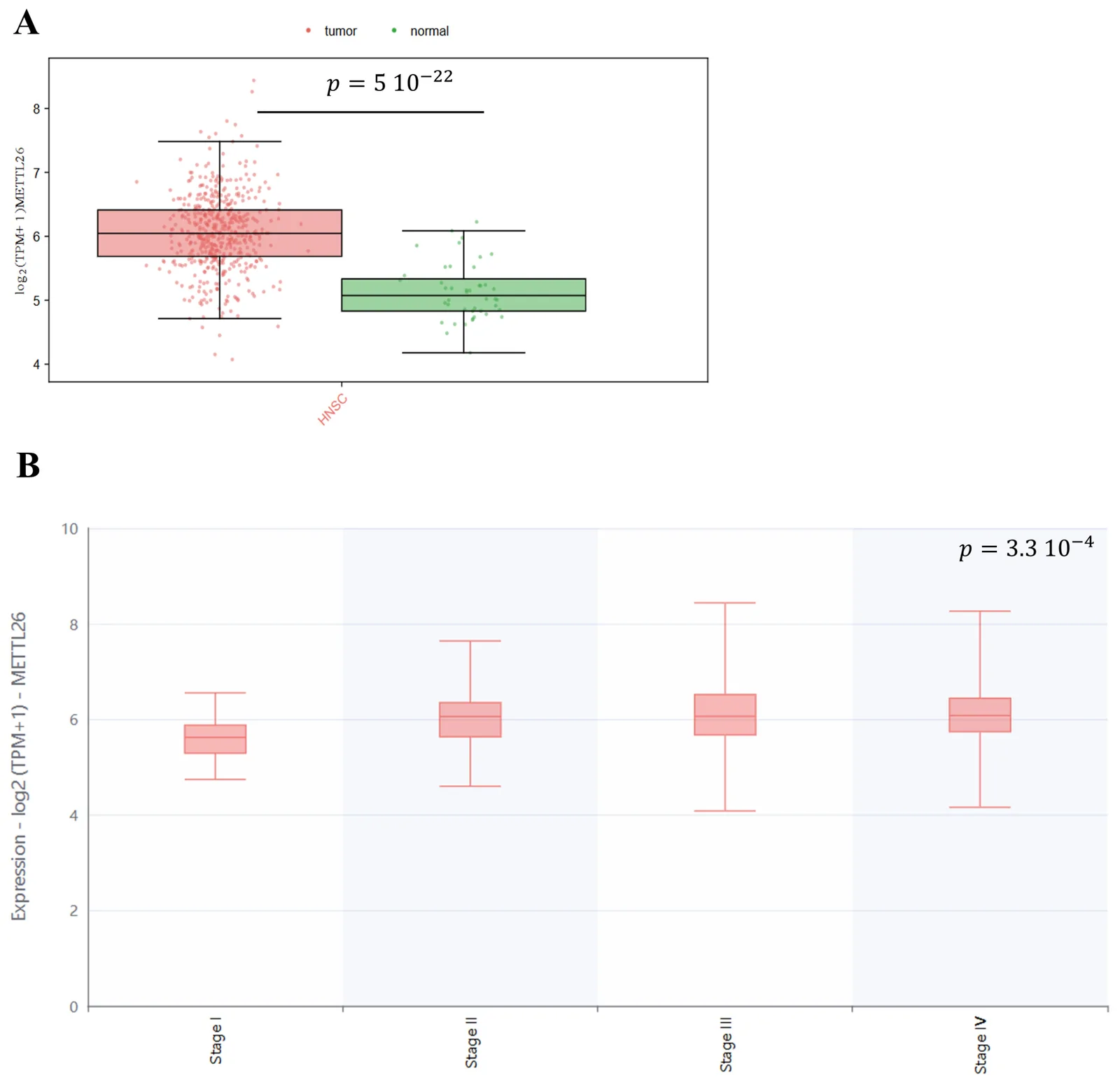
METTL26 expression status in HNSC. (**A**) METTL26 expression levels in TCGA-HNSC tissues (N = 520; red) and non-cancerous tissues (N = 44, green), including TCGA adjacent normal tissues supplemented with GTEx normal tissues, analyzed using GEPIA3. Each point represents an individual sample. (**B**) METTL26 expression according to pathological stage in the TCGA-HNSC cohort: Stage I (N = 27), Stage II (N = 81), Stage III (N = 93), and Stage IV (N = 318). The statistical test performed by GEPIA3 for stage analysis was one-way ANOVA. METTL26 expression is reported as log2(TPM + 1). HNSC, head and neck squamous cell carcinoma; TCGA, The Cancer Genome Atlas; GTEx, Genotype-Tissue Expression; TPM, transcripts per million.

We used GEPIA3, an interactive web server for analyzing the RNA sequencing expression data of normal and cancer tissues, to evaluate variations in METTL26 mRNA expression levels in TCGA-HNSC compared to normal tissues. Interestingly, METTL26 mRNA expression levels were significantly upregulated in TCGA-HNSC compared to normal tissues (Figure 1A).

Furthermore, we evaluated the association between METTL26 mRNA expression levels and pathological stage in the TCGA-HNSC dataset to explore a possible relationship with tumor aggressiveness and disease progression. As shown in Figure 1B, METTL26 expression was significantly associated with tumor stage, with lower expression in stage I tumors and relatively higher levels in stages II–IV, suggesting an association between METTL26 expression and pathological stage, without evidence of a progressive increase across successive stages.

### 3.2. Association of METTL26 Expression with Survival in HNSC

**Figure 2:**
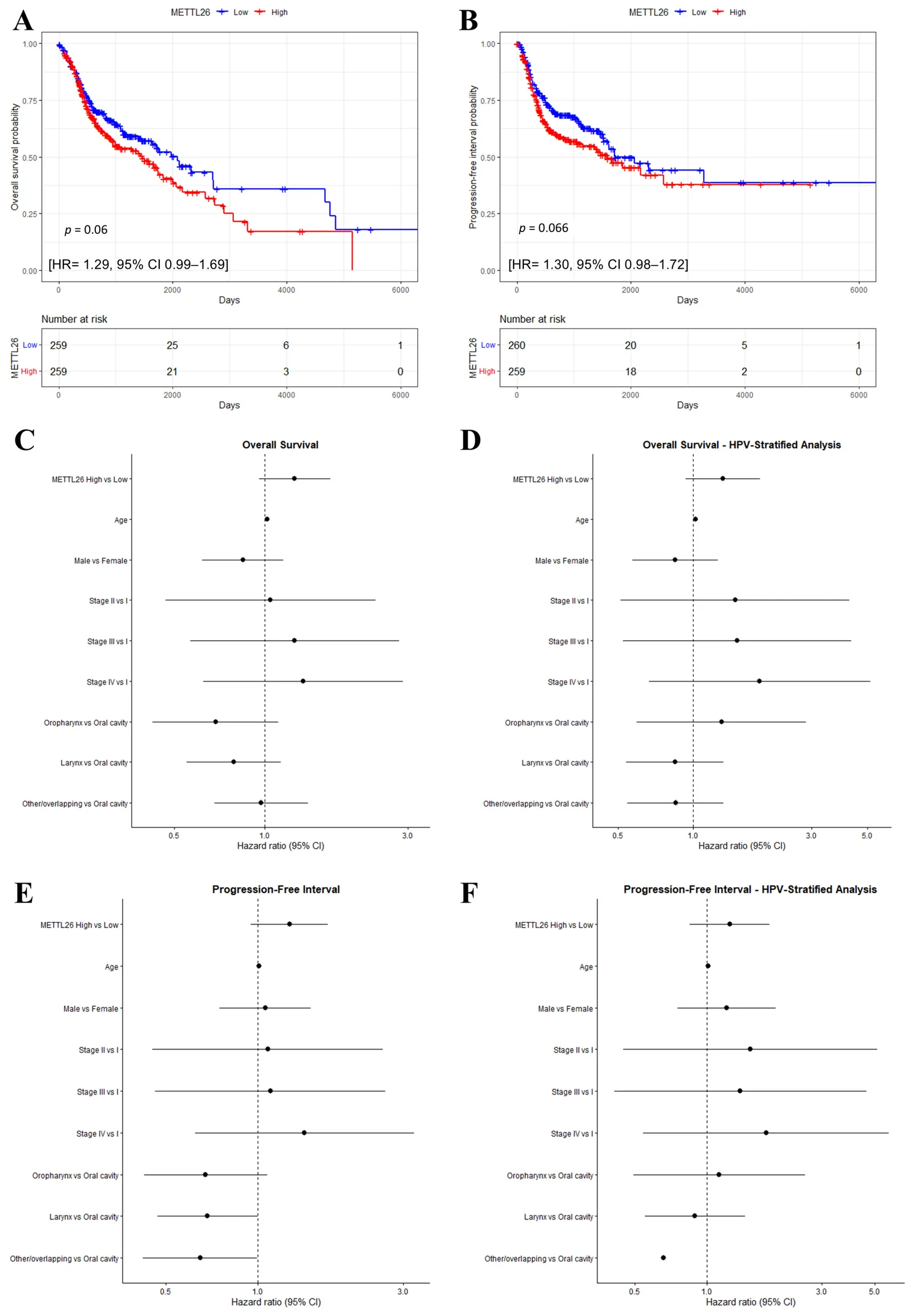
Survival analysis according to METTL26 expression in the TCGA-HNSC cohort. Patients were classified as METTL26-low and METTL26-high using the median METTL26 expression value in the TCGA-HNSC cohort as the cut-off. Kaplan–Meier curves for overall survival (OS) (**A**) and progression-free interval (PFI) (**B**) are shown for METTL26-low (blue) and METTL26-high (red) groups; differences between survival curves were assessed using the log-rank test. Hazard ratios (HRs) and 95% confidence intervals (CIs) were estimated using Cox proportional hazards regression. Forest plots show multivariable Cox models for OS adjusted for age, sex, clinical stage, and anatomical tumor site (**C**). The corresponding HPV-stratified analysis is shown in (**D**). Forest plots show multivariable Cox models for PFI adjusted for age, sex, clinical stage, and anatomical tumor site (**E**). The corresponding HPV-stratified analysis is shown in (**F**). In forest plots, points represent HR estimates and horizontal lines indicate 95% CIs; the vertical dashed line indicates HR = 1. HR, hazard ratio; CI, confidence interval; HPV, human papillomavirus; HNSC, head and neck squamous cell carcinoma; TCGA, The Cancer Genome Atlas.

Kaplan–Meier analysis showed a trend toward poorer overall survival (OS) in patients with high METTL26 expression, although the difference did not reach statistical significance (HR = 1.29, 95% CI 0.99–1.69, *p* = 0.06; Figure 2A). A similar trend was observed for progression-free interval (PFI), with poorer outcomes in METTL26-high tumors, but again without statistical significance (HR = 1.30, 95% CI 0.98–1.72, *p* = 0.066; Figure 2B).

In the multivariable Cox regression analysis adjusted for age, sex, clinical stage, and anatomical site, high METTL26 expression was not independently associated with OS (HR = 1.26, 95% CI 0.96–1.65, *p* = 0.097; Figure 2C). Among the included covariates, increasing age was significantly associated with a higher risk of death (HR = 1.018 per year, 95% CI 1.01–1.03, *p* = 0.007), whereas sex, stage, and anatomical site were not significantly associated with OS.

An additional Cox model was fitted with HPV stratification. In this analysis, the association between high METTL26 expression and OS remained non-significant (HR = 1.32, 95% CI 0.94–1.86, *p* = 0.115; Figure 2D), while age remained significantly associated with survival (HR = 1.023, 95% CI 1.006–1.040, *p* = 0.009).

Similarly, high METTL26 expression was not independently associated with PFI after adjustment for age, sex, stage, and anatomical site (HR = 1.27, 95% CI 0.95–1.70, *p* = 0.106; Figure 2E). In the HPV-stratified analysis, the association was further attenuated (HR = 1.24, 95% CI 0.85–1.83, *p* = 0.266; Figure 2F).

Overall, these analyses indicate that although higher METTL26 expression shows a consistent trend toward poorer OS and PFI, median-dichotomized METTL26 expression does not constitute an independent prognostic factor in the TCGA-HNSC cohort after adjustment for relevant clinicopathological variables.

### 3.3. Association of METTL26 Expression with Cancer-Associated Fibroblasts and Endothelial Cells

**Figure 3:**
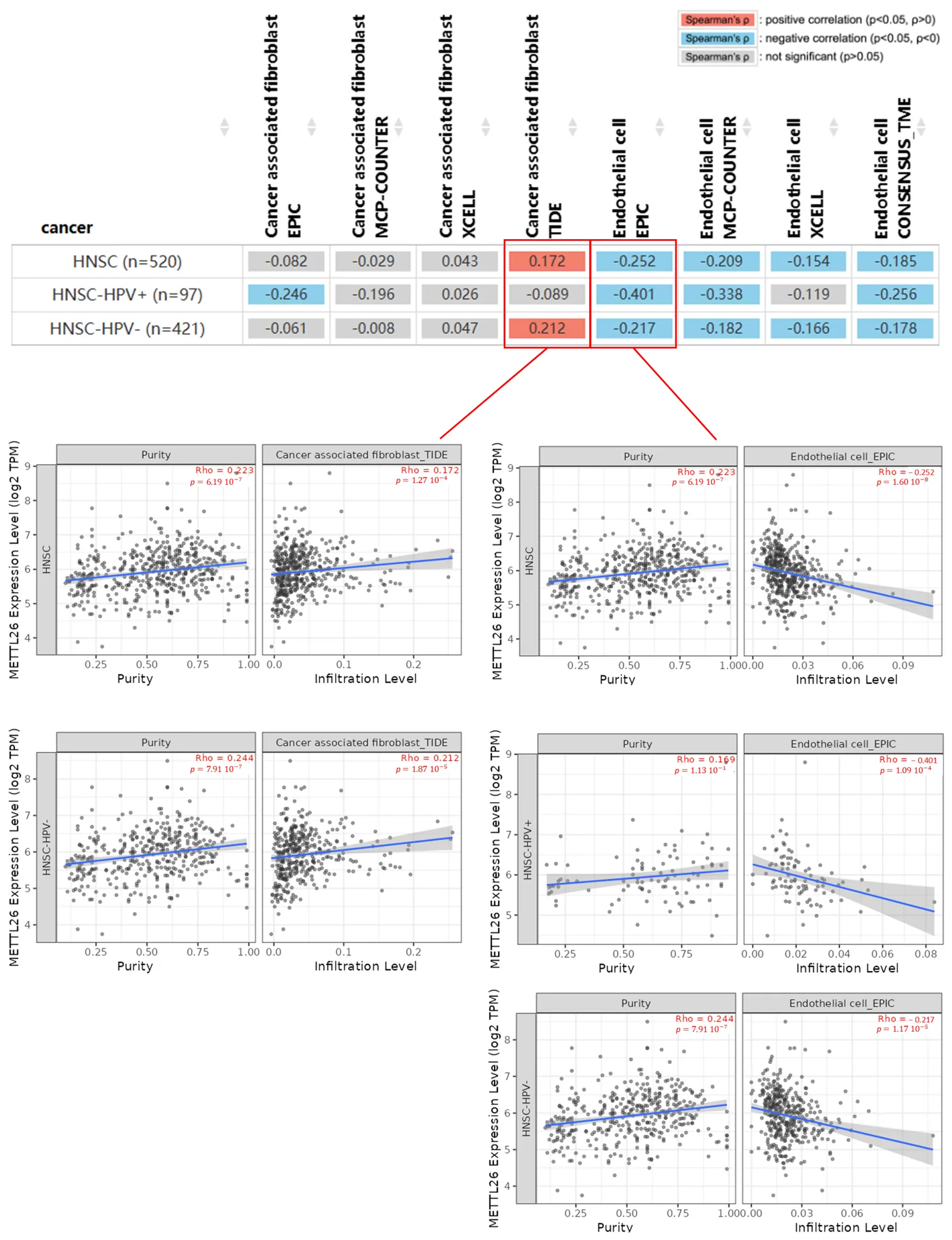
Association of METTL26 Expression with Cancer-Associated Fibroblasts (CAFs) and Endothelial Cells in HNSC within the TCGA dataset using multiple deconvolution algorithms implemented in TIMER3, including TIDE, EPIC, xCell, MCP-counter, and ConsensusTME. Heatmap values represent tumor-purity-adjusted Spearman correlation coefficients (ρ) between METTL26 expression and the estimated abundance of CAFs or endothelial cells. Red cells indicate significant positive correlations (*p* < 0.05), blue cells indicate significant negative correlations (*p* < 0.05), and non-significant correlations are shown in gray. Scatter plots illustrate representative associations obtained using TIDE for CAFs and EPIC for endothelial cells; each point represents an individual tumor sample. A positive correlation between METTL26 expression and CAFs was noted in HNSC and HNSC-HPV- cancers while a significant negative correlation was observed between METTL26 expression and endothelial cells in HNSC, HNSC-HPV- and HNSC-HPV+ cancers. HNSC, head and neck squamous cell carcinoma; HPV, human papillomavirus; CAF, cancer-associated fibroblast; TIDE, Tumor Immune Dysfunction and Exclusion; EPIC, Estimating the Proportions of Immune and Cancer Cells; MCP-counter, Microenvironment Cell Populations-counter; TCGA, The Cancer Genome Atlas.

Since cancer-associated fibroblasts (CAFs) and endothelial cells play an important role in modulating the immune response within the tumor stroma, influencing tumor progression, we used multiple TIMER3-based computational algorithms on the TIMER3.0 web server to evaluate the relationship between METTL26 expression and immune cell infiltration in the TCGA-HNSC dataset. As shown in Figure 3, a positive correlation between METTL26 expression and CAF abundance was found in TCGA-HNSC and HPV-negative HNSC tumors using the TIDE algorithm. In contrast, METTL26 expression showed a negative association with endothelial cell abundance across multiple algorithms, indicating a more consistent cross-method relationship. Therefore, the CAF association should be considered algorithm-dependent and exploratory, whereas the endothelial-cell association appears comparatively more robust.

These findings identify exploratory transcriptomic associations between METTL26 expression and selected stromal components of the tumor microenvironment. It should be noted that, although the use of multiple algorithms provides a broader assessment of immune infiltration and reduces dependence on a single deconvolution method, correlations detected by only one algorithm (or discordant correlations as shown for CAFs by the TIDE algorithm) should be interpreted cautiously and considered more like “hypothesis-generating” rather than a direct functional effect of METTL26 on immune response or immunotherapy efficacy.

### 3.4. Enrichment Analysis of METTL26-Related Genes in HNSC

In order to identify molecular programs associated with METTL26 expression in TCGA-HNSC, we identified the top 100 genes most strongly correlated with METTL26 expression in the TCGA-HNSC dataset using the GEPIA3 “Expression network” module. A fixed top-100 cutoff was used to focus the downstream analyses on the genes showing the strongest co-expression relationships with METTL26 while limiting the contribution of weakly correlated genes. The complete list of the top 100 METTL26-correlated genes is provided in Supplementary Materials. In addition, to characterize biological processes associated with METTL26 expression in TCGA-HNSC, Gene Ontology (GO) and KEGG pathway enrichment analyses were subsequently performed on this co-expression gene set.

**Figure 4:**
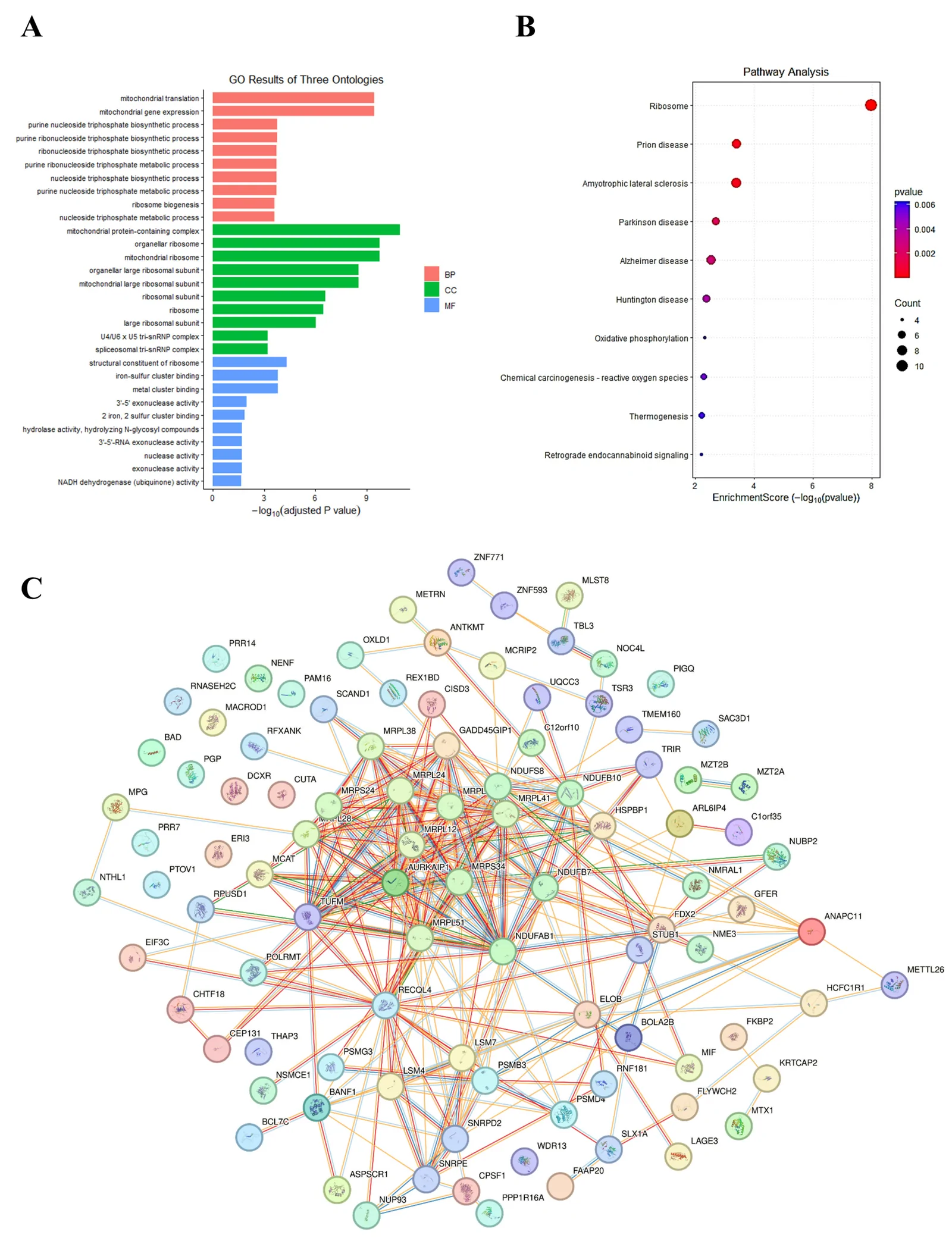
METTL26-related gene enrichment analysis. Gene Ontology (GO) enrichment analysis of METTL26-correlated genes in biological process (BP), molecular function (MF), and cellular components (CC) (**A**). Kyoto Encyclopedia of Genes and Genomes (KEGG) pathway enrichment analysis of the top 100 genes co-expressed with METTL26 obtained by GEPIA3 (**B**). GO and KEGG enrichment analyses were performed using clusterProfiler, with Benjamini–Hochberg correction for multiple testing and significance thresholds of *p* < 0.05 and q < 0.05. In the bubble plot, the enrichment score was expressed as −log10(*p*-value), bubble size represented the number of genes associated with each pathway, and bubble color reflected the corresponding *p*-value. Protein–protein association network of the METTL26-correlated genes generated using STRING with default settings (**C**). Nodes represent proteins encoded by METTL26-correlated genes, whereas edges indicate STRING protein–protein associations; different edge colors correspond to the different evidence channels used by STRING.

As shown in Figure 4A, functional enrichment analysis of these genes revealed a prominent association with mitochondrial and ribosomal functions. In fact, GO analysis showed a strong enrichment in mitochondrial gene expression and mitochondrial translation, together with mitochondrial ribosomal components and ribosome-associated molecular functions. Consistently, KEGG pathway enrichment analysis (Figure 4B) identified “Ribosome” as the most significantly enriched pathway. KEGG analysis also highlighted “oxidative phosphorylation” and “reactive oxygen species-related carcinogenesis” as significantly enriched pathways, while the apparent enrichment of several neurodegenerative disease pathways (see “Amyotrophic lateral sclerosis”, “Parkinson disease”, “Alzheimer disease”, “Huntington disease”) likely reflects the extensive representation of mitochondrial respiratory-chain genes shared by these KEGG signatures rather than a disease-specific association.

Protein-protein interaction analysis performed using STRING further revealed a densely interconnected network characterized by mitochondrial ribosomal proteins (see MRPL proteins) and respiratory-chain components (see NDUFS proteins) (Figure 4C).

Collectively, these findings indicate that METTL26 expression in TCGA-HNSC is associated with co-expression programs related to mitochondrial protein synthesis, oxidative metabolism, and redox homeostasis.

### 3.5. Correlation of METTL26 with Therapy-Response-Related Genes

**Figure 5:**
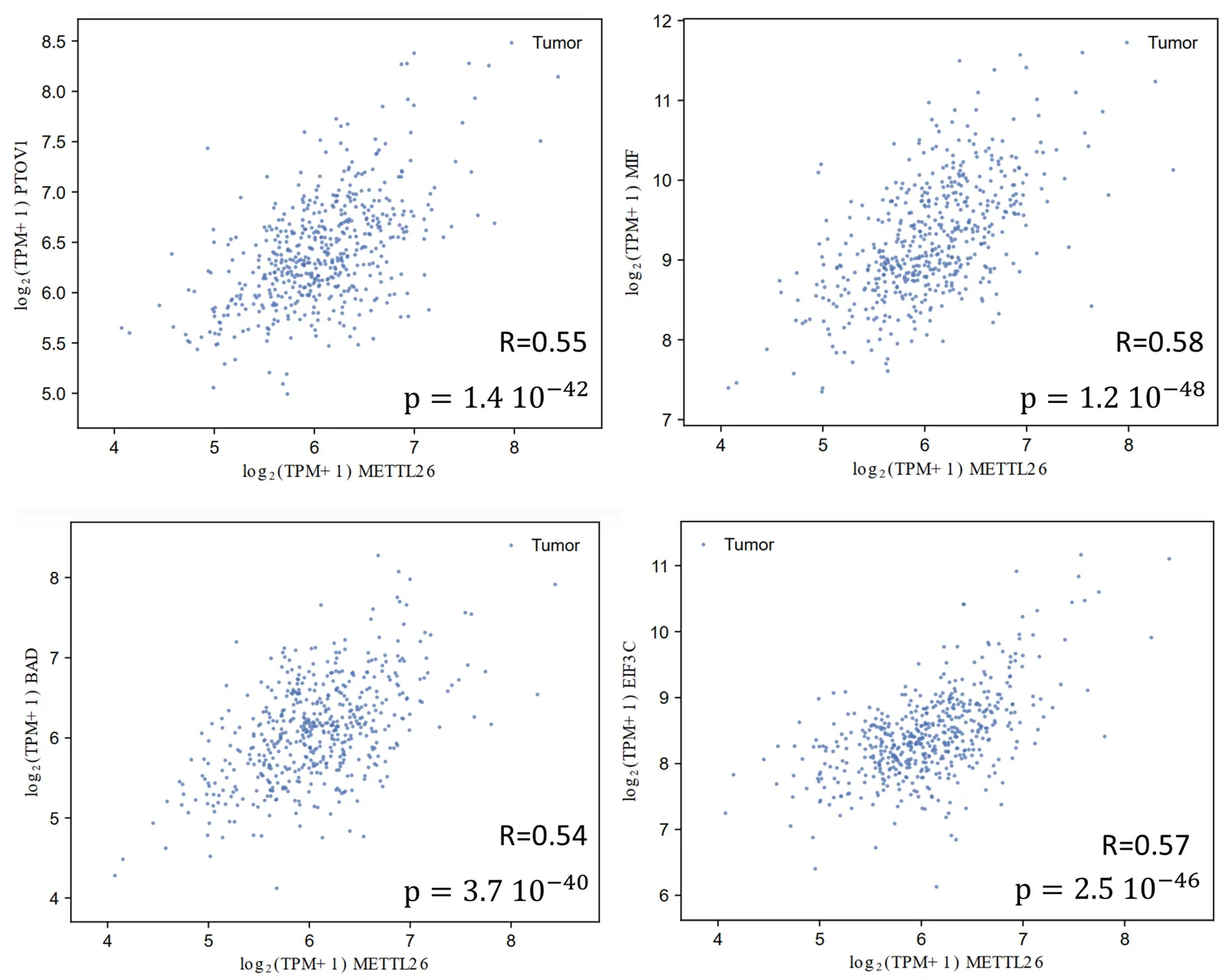
Correlation analysis between METTL26 and PTOV1, MIF, BAD, and EIF3C in TCGA-HNSC tumor samples using GEPIA3. Scatter plots show the relationship between METTL26 expression and prostate tumor overexpressed 1 (PTOV1), macrophage migration inhibitory factor (MIF), BCL2-associated agonist of cell death (BAD), and eukaryotic translation initiation factor 3 subunit C (EIF3C) expression in TCGA-HNSC tumor samples, as determined using GEPIA3. Each point represents an individual tumor sample. METTL26 and target-gene expression values are reported as log2(TPM + 1). The correlation coefficient (R) and corresponding *p* value are reported in each panel.

As shown in Figure 5, among the genes most strongly correlated with METTL26, several genes, including PTOV1, MIF, BAD, and EIF3C, have previously been linked to therapy response in the literature [26,27,28,29]. These post hoc observations are exploratory and hypothesis-generating and should not be interpreted as evidence of a predefined therapy-response signature or of a direct role of METTL26 in treatment resistance.

### 3.6. Validation of METTL26 Expression in Two Independent Cohorts

To validate the METTL26 expression results obtained in TCGA-HNSC in independent cohorts, we used two GEO datasets (GSE37991 and GSE30784).

**Figure 6:**
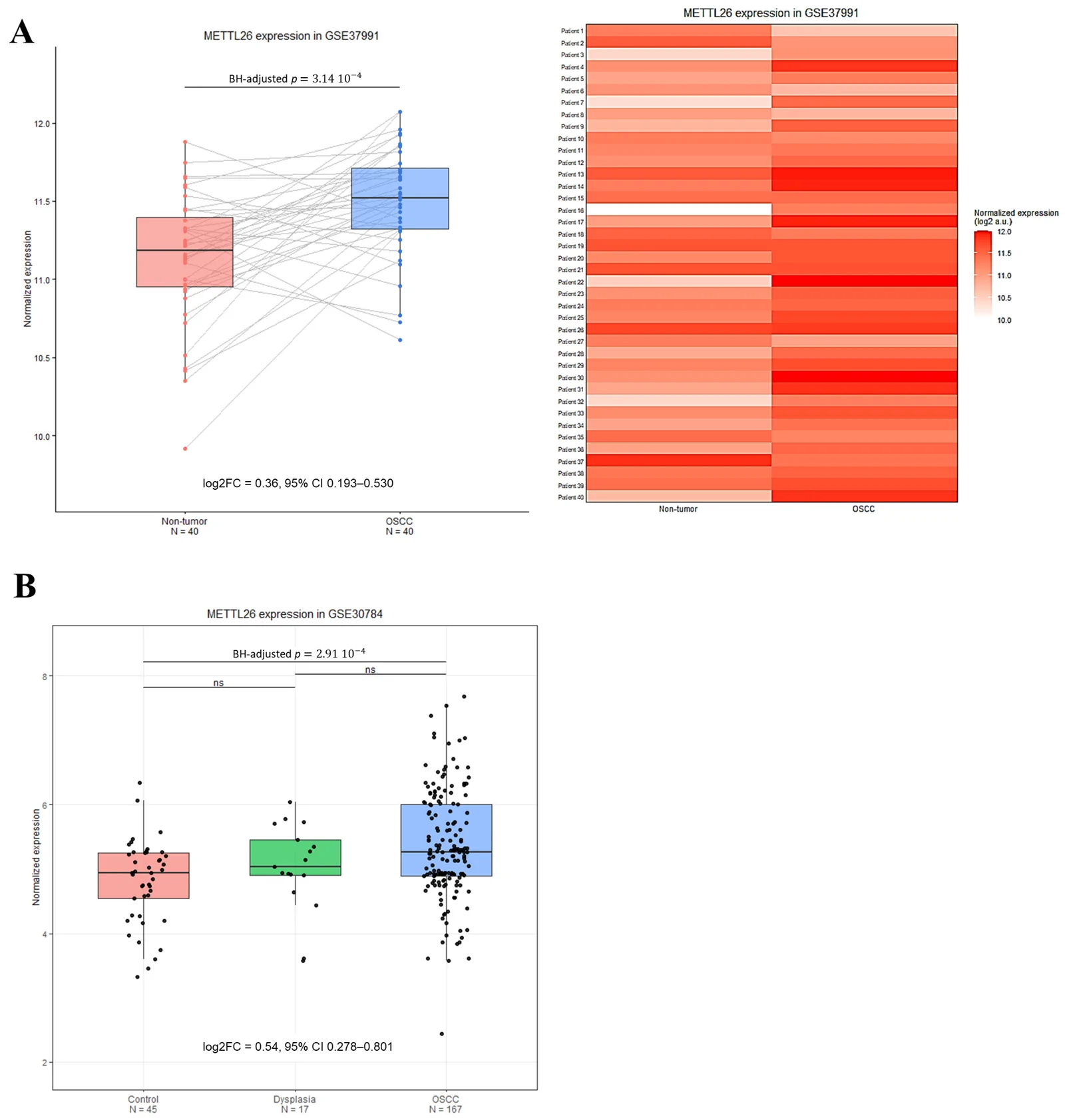
Independent validation of METTL26 expression in two OSCC cohorts. (**A**) METTL26 expression in the GSE37991 cohort, comprising paired OSCC samples (N = 40, blue) and adjacent non-tumor tissues (N = 40, red) from the same patients. Expression values were quantile-normalized and log2-transformed. In the boxplot, gray lines connect paired samples from the same patient. The adjacent heatmap shows METTL26 expression for each matched patient pair, with increasing color intensity indicating higher expression. Differential expression was assessed using limma with patient identity and tissue status included in the linear model to account for the paired design. (**B**) METTL26 expression in the independent GSE30784 cohort comprising normal oral tissues (N = 45; red), oral dysplasia samples (N = 17; green), and OSCC samples (N = 167; blue). Dots represent individual samples. Differential expression was evaluated using limma with tissue category as a three-level factor followed by pairwise contrasts and Benjamini–Hochberg correction. CI, confidence interval; BH, Benjamini–Hochberg; ns, not significant.

The independent GSE37991 cohort consists of 40 paired OSCC and adjacent non-tumor tissues. In this cohort, we found a statistically significant increase in METTL26 expression in tumor samples compared to the adjacent non-tumor tissues (log2FC = 0.36, 95% CI 0.193–0.530, BH-adjusted *p* = 3.14 × 10^−4^) (Figure 6A). Although some inter-patient variability was observed, most paired samples showed higher METTL26 expression in OSCC, independently confirming its upregulation in OSCC. These findings were further confirmed by the paired heatmap analysis (Figure 6A).

To further validate our results, we used another independent cohort (GSE30784). This cohort consists of 45 normal tissues, 17 dysplastic tissue samples, and 167 OSCC tissues. In this cohort, METTL26 expression was significantly increased in OSCC compared with normal oral tissues (log2FC = 0.54, 95% CI 0.278–0.801, BH-adjusted *p* = 2.91 × 10^−4^), whereas no statistically significant differences were found between dysplastic lesions and either normal tissues or OSCC (Figure 6B).

Taken together, these data independently confirmed the differential-expression finding, namely METTL26 upregulation in OSCC compared with non-tumor tissues. The other TCGA-HNSC-derived clinical and molecular associations were not evaluated in these validation cohorts.

## 4. Discussion

METTL26 belongs to the METTL family of S-adenosylmethionine-dependent methyltransferases, several members of which have emerged as important regulators of cancer-related processes [16,17,18,19]. METTL-dependent mechanisms have also attracted increasing attention in HNSCC [20]. Nevertheless, METTL26 remains one of the least characterized members of this family. The present study provides a focused characterization of METTL26 in TCGA-HNSC, with particular relevance to OSCC, extending previous pan-cancer observations on this poorly characterized METTL family member. In this study, the TCGA-HNSC cohort was used as a discovery dataset to identify the initial METTL26-associated molecular and clinical signals in a large cohort of HNSCC tumors, whereas the expression findings were subsequently validated in two independent OSCC-specific cohorts.

Previous evidence regarding METTL26 in cancer was provided by Campeanu et al., who investigated METTL26 as part of a multi-omics analysis of 34 METTL family members across human malignancies [22]. Their study identified METTL26 dysregulation in selected tumor types, reported clinical associations, and suggested a possible involvement of METTL26-associated proteins in vacuolar transport. The present study therefore extends these previous pan-cancer observations through a disease-focused investigation in HNSC/OSCC, including analyses of pathological stage, survival, tumor microenvironment composition and co-expression programs, together with independent validation of METTL26 upregulation in two OSCC cohorts.

Our findings showed that, in the TCGA-HNSC cohort, METTL26 expression was significantly upregulated in cancer tissues compared to normal tissues. Moreover, METTL26 expression showed a significant association with pathological stage, whereas the association with OS and PFI did not reach statistical significance. However, because these analyses were performed in the complete TCGA-HNSC cohort, OSCC-specific cohorts with appropriate adjustment for stage, anatomical site, treatment, and other clinicopathological variables will be required to determine whether METTL26 has independent prognostic value in oral cancer.

However, these findings support further investigation of METTL26 in the context of HNSCC biology.

The association between METTL26 and stromal components of the tumor microenvironment represents another interesting finding. In fact, TIMER3 analysis showed a positive relationship between METTL26 expression and CAF abundance in HNSC and HPV-negative TCGA-HNSC using the TIDE algorithm. This association is noteworthy because CAF accumulation has been associated with aggressive clinicopathological features and unfavorable outcomes in OSCC [30]. However, the CAF association detected in the present study was not consistently reproduced across the different deconvolution algorithms and should therefore be considered exploratory. Conversely, the inverse correlation between METTL26 expression and endothelial cell abundance was observed more consistently across several algorithms. These results may highlight specific differences in vascular abundance, stromal composition, or angiogenic activity in TCGA-HNSC. However, even in this case, bulk transcriptomic deconvolution cannot establish a direct biological interaction between METTL26 and endothelial cells, highlighting the need for single-cell and/or spatial transcriptomic approaches, together with immunohistochemical localization studies of METTL26 in tumor and stromal compartments, to clarify these relationships.

GO analysis highlighted a strong association between METTL26 expression and genes involved in mitochondrial and ribosomal functions, while KEGG analysis identified ribosome, oxidative phosphorylation and reactive oxygen species-related carcinogenesis among the significantly enriched pathways. Consistently, the interaction network contained several mitochondrial ribosomal proteins and respiratory-chain components. These findings are consistent with previous studies reporting alterations in mitochondrial protein synthesis and localization [31,32] and an association between an oxidative phosphorylation-related gene signature and OSCC recurrence and poor survival [33]. Indeed, accumulating evidence indicates that mitochondrial metabolic reprogramming and metabolic plasticity between glycolysis and oxidative phosphorylation contribute to OSCC growth, invasion, recurrence, and therapeutic resistance [33,34,35].

Taken together, these enrichment analyses indicate that genes co-expressed with METTL26 are associated with mitochondrial protein synthesis, oxidative metabolism, and redox-related pathways. However, these transcriptomic associations do not demonstrate that METTL26 directly regulates mitochondrial respiration, ROS production, mitochondrial translation, or metabolic reprogramming. Functional studies based on METTL26 silencing and/or overexpression will therefore be required to establish whether these associations have biological relevance.

Another notable finding was the positive correlation between METTL26 and PTOV1, MIF, BAD, and EIF3C, genes involved in biological processes that may influence tumor response to chemotherapy and radiotherapy [26,27,29]. Collectively, these correlations suggest that METTL26 expression may occur within a broader transcriptional program involving stress adaptation, apoptosis, protein synthesis, and therapeutic response. However, the co-expression of these genes with METTL26 does not demonstrate that METTL26 directly regulates these genes or causes chemo- or radioresistance. These associations should be considered hypothesis-generating and do not demonstrate that METTL26 directly regulates therapy resistance. Thus, gain- and loss-of-function studies involving METTL26 overexpression or silencing, followed by cisplatin and irradiation sensitivity assays, will be required to establish whether this association has functional relevance.

The clinical associations observed in TCGA-HNSC provide a rationale for further investigating METTL26 as a candidate expression marker in HNSCC.

Following the analysis of TCGA-HNSC, which was used as the discovery dataset, METTL26 upregulation was reproduced in two independent OSCC datasets (GSE37991 and GSE30784), supporting the relevance of the initial HNSC-derived observation to oral carcinogenesis, as OSCC represents the predominant malignant histological type of the oral cavity [7]. Importantly, these independent cohorts validate the differential-expression phenotype only; the associations with pathological stage, survival, tumor microenvironment composition, mitochondrial pathways, and therapy-response-related genes require independent validation in ad hoc studies.

The analysis performed in the validation cohort GSE30784 did not show significant differences in METTL26 expression between dysplastic lesions and either normal or OSCC tissues, suggesting that the increase in METTL26 expression is not progressive throughout oral epithelial transformation. Thus, the available data do not support a progressive or stepwise increase in METTL26 expression throughout oral epithelial transformation.

**Figure 7:**
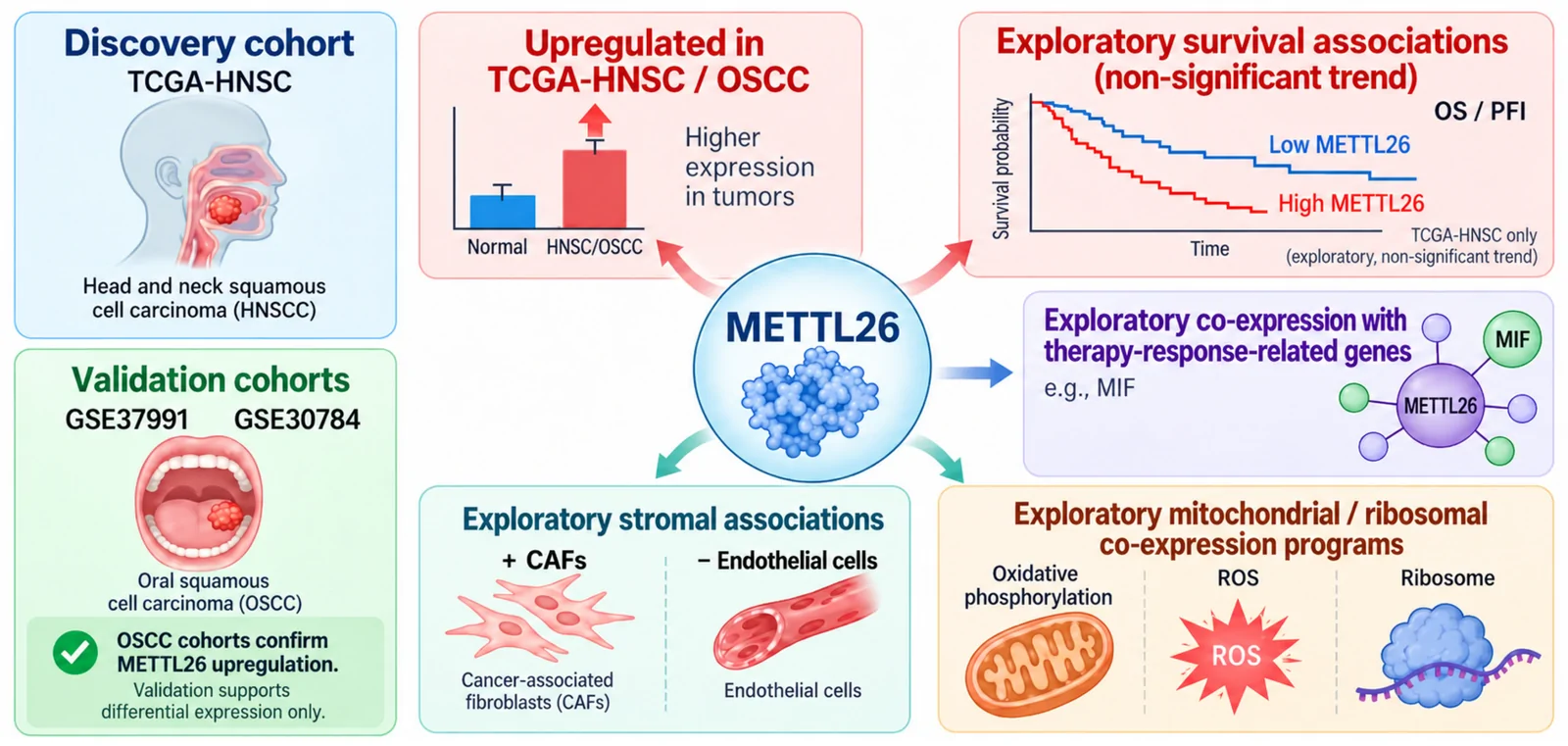
Integrated overview of METTL26 alterations and associated molecular features in HNSC/OSCC. METTL26 expression was evaluated in the TCGA-HNSC discovery cohort and validated in the independent GSE37991 and GSE30784 OSCC cohorts, confirming increased expression in tumor tissues. Higher METTL26 expression showed a trend toward poorer overall survival (OS) and progression-free interval (PFI). Computational analyses further associated METTL26 with increased cancer-associated fibroblast (CAF) infiltration, reduced endothelial cell representation, co-expression with therapy-response-related genes such as MIF, and mitochondrial/ribosomal pathways, including oxidative phosphorylation and reactive oxygen species (ROS)-related processes. Only METTL26 upregulation was independently validated in the external OSCC cohorts; the survival, stromal, pathway, and therapy-response-related associations derive from exploratory analyses of TCGA-HNSC and require independent validation.

METTL26 overexpression in OSCC tissues was also confirmed in the GSE37991 cohort. This cohort includes paired tumor and adjacent non-tumor tissues from the same patients, reducing the influence of inter-individual variability. The consistency of these findings across cohorts generated using different microarray platforms significantly increases confidence that METTL26 upregulation is not restricted to the TCGA-HNSC dataset. However, further studies are required to validate METTL26 expression at the protein level before considering METTL26 a clinically relevant OSCC biomarker. A schematic representation of the main findings of this study is shown in Figure 7.

## 5. Limitations

Several limitations should be acknowledged. First, the analyses were based on retrospective, publicly available bulk transcriptomic datasets and therefore cannot establish causality or resolve cell-type-specific expression patterns. Second, although multivariable survival analyses were performed in TCGA-HNSC, independent OSCC-specific survival validation is lacking. Third, METTL26 expression has not yet been validated at the protein level, and no diagnostic-performance analysis was performed to assess its sensitivity, specificity, or discriminative ability. In addition, the functional enrichment analyses were based on the top 100 METTL26-correlated genes, an arbitrary threshold that may influence the resulting pathway representation. Finally, no functional experiments were performed in the present study to define METTL26 activity, substrates, or biological effects in OSCC cells.

## 6. Conclusions

This study identifies METTL26 as a consistently upregulated gene in TCGA-HNSC and in two independent OSCC cohorts, supporting further investigation of its potential value as an expression marker. The associations with pathological stage, survival, tumor microenvironment composition, mitochondrial/redox-related pathways, and therapy-response-related genes remain exploratory and were not independently validated in the external OSCC cohorts. Notably, the direct functional role of METTL26 in HNSCC and OSCC has not yet been established, consistent with its current classification as a Tdark protein [36]. Further OSCC-specific clinical validation and functional studies are therefore required to define its biological and clinical relevance.

## Figures and Tables

**Table 1:** Characteristics of the GEO datasets used for METTL26 expression validation.

	GSE30784	GSE37991
PLATFORM	Affymetrix Human Genome U133 Plus 2.0 Array	Illumina HumanRef-8 v3.0 Expression BeadChip
GPL ACCESSION	GPL570	GPL6883
TOTAL SAMPLES	229	80
OSCC SAMPLES	167	40
CONTROL SAMPLES	45 controls	40 adjacent non-tumor tissues
DYSPLASIA SAMPLES	17	Not included

## Data Availability

The publicly available datasets analyzed in this study were obtained from The Cancer Genome Atlas (TCGA-HNSC) and the Gene Expression Omnibus (GEO; accession numbers GSE30784 and GSE37991). To ensure transparency and facilitate independent reproducibility, the R script used for the bioinformatic and statistical analyses, together with the corresponding input tables and supporting files, has been provided as Supplementary Materials. These resources allow independent verification and reproduction of the R-based analyses presented in the manuscript. Additional analyses performed using GEPIA3, TIMER3, and STRING can be independently replicated using the publicly accessible platforms and the methodological parameters described in the Materials and Methods section.

## Supplementary Materials

The following supporting information can be downloaded at: https://www.mdpi.com/article/10.3390/medsci14060674/s1, Table S1: GO, BP, CC and MF results; Table S2: KEGG enrichment results with symbols; Table S3: Top 100 METTL26-related genes; R Script file: Script file of METTL26 analysis performed in R software.

## Abbreviations

The following abbreviations are used in this manuscript:

ATP	Adenosine Triphosphate
BAD	BCL2 Associated Agonist of Cell Death
BH	Benjamini–Hochberg
BP	Biological Process
CAF	Cancer-Associated Fibroblast
CC	Cellular Component
DNA	Deoxyribonucleic Acid
EIF3C	Eukaryotic Translation Initiation Factor 3 Subunit C
EPIC	Estimating the Proportions of Immune and Cancer Cells
FAAP20	Fanconi Anemia Core Complex Associated Protein 20
GEO	Gene Expression Omnibus
GEPIA3	Gene Expression Profiling Interactive Analysis, Version 3
GO	Gene Ontology
GPL	GEO Platform
GSE	GEO Series
HNSC	Head and Neck Squamous Cell Carcinoma
HPV	Human Papillomavirus
HPV16	Human Papillomavirus Type 16
IRCCS	Istituto di Ricovero e Cura a Carattere Scientifico
KEGG	Kyoto Encyclopedia of Genes and Genomes
MCP-counter	Microenvironment Cell Populations-counter
METTL	Methyltransferase-Like
METTL26	Methyltransferase-Like 26
MIF	Macrophage Migration Inhibitory Factor
MRPL	Mitochondrial Ribosomal Protein, Large Subunit
mRNA	Messenger RNA
NDUFS	NADH:Ubiquinone Oxidoreductase Core Subunit S
OS	Overall Survival
OSCC	Oral Squamous Cell Carcinoma
PFI	Progression-Free Interval
PTOV1	Prostate Tumor Overexpressed 1
RECQL4	RecQ Like Helicase 4
RNA	Ribonucleic Acid
ROS	Reactive Oxygen Species
STRING	Search Tool for the Retrieval of Interacting Genes/Proteins
TCGA	The Cancer Genome Atlas
TIDE	Tumor Immune Dysfunction and Exclusion
TIMER3	Tumor Immune Estimation Resource, Version 3
TNM	Tumor–Node–Metastasis
TPM	Transcripts Per Million
xCell	xCell Cell-Type Enrichment Analysis
